# PVDF Nanofiber Sensor for Vibration Measurement in a String

**DOI:** 10.3390/s19173739

**Published:** 2019-08-29

**Authors:** Rahul Kumar Singh, Sun Woh Lye, Jianmin Miao

**Affiliations:** School of Mechanical and Aerospace Engineering, Nanyang Technological University, 50 Nanyang Ave, Block N3, Nanyang Ave, Singapore 639798, Singapore

**Keywords:** PVDF nanofiber sensor, sensor on string, vibration sensor, strain sensor, impact measurement

## Abstract

Flexible, self-powered and miniaturized sensors are extensively used in the areas of sports, soft robotics, health care and communication devices. Measurement of vibration is important for determining the mechanical properties of a structure, specifically the string tension in strings. In this work, a flexible, lightweight and self-powered sensor is developed and attached to a string to measure vibrations characteristics in strings. Electrospun poly(vinylidene) fluoride (PVDF) nanofibers are deposited on a flexible liquid crystal polymer (LCP) substrate for the development of the sensor. The electrospinning process is optimized for different needle sizes (0.34–0.84 mm) and flow rates (0.6–3 mL/h). The characterization of the sensor is done in a cantilever configuration and the test results indicate the sensor’s capability to measure the frequency and strain in the required range. The comparison of the results from the developed PVDF sensor and a commercial Laser Displacement Sensor (LDS) showed good resemblance (±0.2%) and a linear voltage profile (0.2 mV/με). The sensor, upon attachment to a racket string, is able to measure single impacts and sinusoidal vibrations. The repeatability of the results on the measurement of vibrations produced by an impact hammer and a mini shaker demonstrate an exciting new application for piezoelectric sensors.

## 1. Introduction

The advent of flexible electronics and Internet of Things (IOT) has led a surge in the development of smart devices in different facets of life. The applications of these devices cover key areas such as healthcare, sports, robotics and communication devices [1,2,3,4]. The heart of these smart devices are sensors that can measure properties like strain and vibration [5,6]. Vibration is an important mechanical phenomenon that can be generated by a disturbance produced in a mechanical structure. The vibration produced can be desirable as in the case of musical notes and energy harvesters or undesirable as in the case of bridge or building oscillations [7,8]. Numerous sensors have been developed to measure vibrations in structures such as tall buildings, machine parts, human motion monitoring and cantilever structures [9,10,11]. Although in the past, sensors have been developed for vibration measurement, to the best knowledge of the authors, the majority of them are designed for flat surfaces and not for small curved surfaces, for example, strings.

Strings are important components of many natural and artificial structures around us, for example, human ligaments, cable suspension bridges, sports rackets, musical instruments, humanoid robots and fabrics. Measurement of impact and vibrations in strings are important because they can provide information about the mechanical properties of the strings such as the string tension. Taking an example of tennis rackets, larger vibrations are produced when the impact of the ball on the racket is near the center than an off-center impact. The sweet spot which is the desired impact point for a tennis player produces the least vibration and most power in the shot [12]. Hence, the measurement of vibrations from the string can help improve the player performance in tennis by providing the player with information on location of the ball hit on the racket. Grimes [13] established a relationship between the frequency of a single guitar string and string tension upon the impact of a force. This tension can be determined through the measurement of frequency of vibration during the plucking action of a guitar string. The measurement of the frequency of vibrations has been utilized to determine the string tension in the tuning of guitar strings [14] as well as estimating the cable tension in bridge structures by Wei-Xin [15].

There have been various research works involving sensors used to determine strain and frequency of vibration. Traditionally, piezoresistive films [16,17] have been used to measure vibrations in several applications. Also, optics-based techniques such as fiber Bragg gratings and optical fiber cantilevers have been developed for the measurement of vibration [18,19]. However, these sensors require an external power source and hence are difficult to miniaturize. Another set of sensors relates to piezoelectric sensors which have gained popularity mainly for mechanical energy harvesting applications. These sensors can be self-powered during operation and make use of inorganic nanofibers which have outstanding piezoelectric properties. They are however found to be brittle and hence lack flexibility [20,21,22], so their use for measuring vibration on a flexible and elastic surface such as string can be restrictive. In more recent works, piezoelectric polymers like poly(vinylidene) fluoride (PVDF) are used for strain sensing and monitoring of composite structures and wearable devices [23,24,25]. These PVDF films are found to be sensitive and flexible. Hence, they are suitable for applications with larger strain values. The use of these films is however limited as they need to be electrically poled before they can be adopted [26,27,28]. Piezoelectric nanofibers made from polymer materials with the use of electrospinning such as PVDF do not have such limitations. They do not need additional poling as it is achieved during the electrospinning process itself and they are also more flexible and sensitive than the film. Also, PVDF nanofibers have an elongation to break value of 17% which is significantly larger than the highest strain found in films [29,30]. Hence, they have been used in fabrication of a plethora of sensors like biosensors, sensors for robotics, wearable sensors, tactile sensors, electronic yarns and pressure sensors [31,32,33,34,35,36]. Their superior properties have been utilized to fabricate sensors that can measure vibration signals such as those generated by a force of impact or by acoustic waves [37,38,39]. By using PVDF nanofibers generated by electrospinning, small, lightweight and self-powered sensors could be developed and placed on strings for high strain and accurate vibration measurements.

Amongst the studies pertaining to sensors utilized to measure string properties, a few studies taking a racket string as an example have focused on developing sensors for determining strain and frequency. One commercial device by Stringmeter [40] involves making use of two metal rods to provide a torque to the string at the point of overlap. The device has a spring inside which measures the resistance of the string relative to the torque which in turn is used to determine the string tension. Another device by the name of ERT 300 [41] measures the vibration response in a string by impacting the string with a metal object. By monitoring the vibration response, it seeks to predict the string tension via an established relationship between these two parameters. In another work by Cross et al. [42], a set of piezo discs was used to measure the vibration response of a racket string. Piezo disks were attached to the strings and generated an electrical output upon impact on the string. Through an established relationship between string tension and vibration frequency, the string tension would be obtained. This approach is however not very accurate as the mass of the piezo discs is needed to be compensated by adding a factor to the frequency values obtained. It was found that the string tension values over time can vary by as much as 40% from the time of stringing. More recently, Valentine [43] used a linear variable displacement transducer (LVDT) to determine the displacement of the strings in a tennis racket upon being impacted by a tennis ball. The work involved attaching LVDT sensors to the strings of a tennis racket with a tennis ball being tossed from a ball launcher. Preliminary results reported that the LVDT sensors can measure the deflection of the strings as well as the frequency of vibration of the strings upon impact of the tennis ball. In another work, Hennig [44] made use of a set of steel wires wound around the strings to measure impact location of the ball on a tennis racket. The change in the electrostatic charge in the steel wires was measured upon impact of the ball on the racket strings. Based on the measured charge distribution profile in each of the individual wires, the approach seeks to predict the location of the impact force. Although there have been attempts to develop sensors to measure different string characteristics, in most cases, the sensor mass or the attached mass is large and hence changes the mechanical properties of the string. Also, these sensors require an external voltage source to operate. These limitations would make it difficult to miniaturize the sensor for practical application and hence affect the performance of the string.

This research focuses on the development of a self-powered, flexible and lightweight sensor that can be attached to a string. The sensor should be able to withstand high impact forces and yet be able to measure strain and vibration characteristics in the desired application range. In the present study, a vibration sensor made from electrospun PVDF nanofibers has been developed for the measurement of vibration on a string. Improved β-phase is obtained by varying the needle size and flow rate in the electrospinning of the PVDF nanofibers which in turn is known to improve the voltage output of the sensor. The sensor is tested in cantilever mode first for the required range of vibration frequencies and strain. The performance of the sensor is measured against a commercial Laser displacement sensor (LDS) and the sensor shows correct and repeatable performance. A linear voltage-strain relationship is obtained in the required range of strain. The sensor is attached to the string and tested for vibration measurement. The sensor shows accurate and repeatable readings on single impact and sinusoidal displacement of the string.

## 2. Materials and Methods

**Figure 1:**
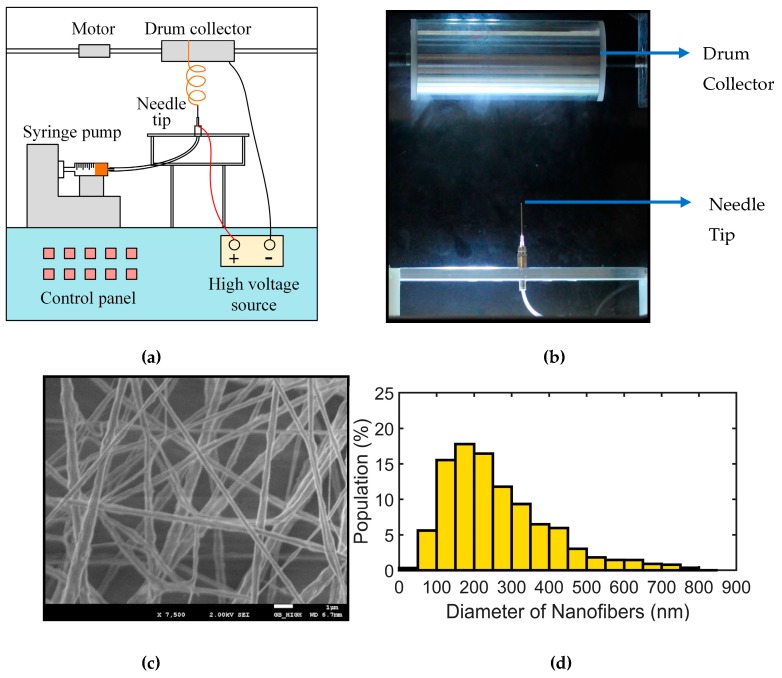
(**a**) Schematic of the electrospinning system used for formation of PVDF nanofibers; (**b**) Enlarged view of the needle and the drum collector arrangement of the electrospinning system; (**c**) SEM image of a sample of PVDF nanofibers collected from electrospinning; (**d**) Histogram of the nanofiber diameters present in the sample.

PVDF nanofibers are formed using an electrospinning set-up with a needle and a rotating drum collector that is shown in Figure 1a. The set-up is a commercial electrospinning system (NE-300, Innovenso Technologies, Boston, MA, USA) which allows for better control of humidity and temperature compared to an open system. To form the nanofibers, 2.0 g of PVDF powder, with a molecular weight of 534,000, is first dissolved in 4 mL of acetone and 6 mL of dimethylformamide (DMF) (chemicals purchased from Sigma Aldrich, St. Louis, MO, USA) to obtain a 20% weight by volume solution. The above composition is selected as it has been shown to produce uniform nanofibers without beads and a ratio of 6:4 of DMF and acetone produces a smoother flow compared to one with more acetone. This is due to the higher evaporation of acetone in the latter case which causes the needle to be clogged [45]. The composition is stirred magnetically for 3 h on a hot plate to obtain a clear, transparent and viscous solution. The solution is extracted into 10 mL syringes for use in the electrospinning process. Different sized needles are used as nozzles, with the internal diameter varying from 0.34 to 0.84 mm. Also, flow rates ranging from 0.6 mL/h to 3.0 mL/h are used to see their effect on the electrospun nanofibers. The collector consists of a 10 cm diameter rotating drum with aluminum foil wrapped around it for deposition of the nanofiber samples. As can be seen from Figure 1a, the nozzle is connected to the positive terminal with the collector connected to the ground of the machine. For the formation of nanofibers, the rotational speed of the drum collector is set at 1000 rpm to facilitate adherence of nanofibers to the drum. A digital picture with an enlarged view of the needle and the drum collector used for electrospinning has been shown in Figure 1b.

The voltage applied is kept constant at 15 kV for all the experiments as the electrospinning process is found to be most stable at this voltage. The flow rate of the solution is kept constant at 2.0 mL/h which enables a smooth formation of the nanofibers. The distance between the needle tip and the collector is set at 150 mm. To ensure the quality of the nanofiber samples, scanning electron microscopy is used to examine the morphology of the nanofibers generated. The sample is prepared at a flow rate of 2.0 mL/h and a needle size of 0.34 mm. The SEM image in Figure 1c shows a generally uniform electrospun sample. Based on the SEM image of a nanofiber sample, most fibers are found between 150–300 nm size after 30 min of electrospinning time as shown in the histogram in Figure 1d. The mean size of the nanofibers is found to be (218 ± 53) nm and the nanofibers are free of beads.

**Figure 2:**
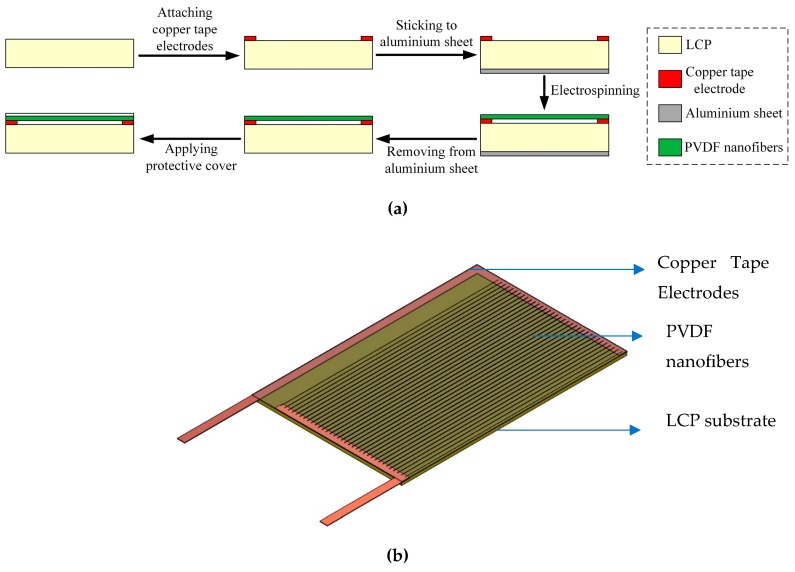
Development of the PVDF nanofiber sensor. (**a**) Schematic of the fabrication steps of the sensor; (**b**) Schematic of the final developed sensor.

The sensor is composed of liquid crystal polymer (LCP) as the substrate, a set of copper electrode tapes and PVDF nanofibers as the sensing material. LCP is chosen as the substrate since it can be of low thickness, lightweight and highly bendable [46,47]. It has good chemical resistance and hence can maintain its properties in harsh environments [48,49,50]. It also has good mechanical robustness and can take large impact force [50]. In the current work, 50 μm thick LCP was bought from Roger Corporation (Chandler, AZ, USA). The set of electrodes for the sensor are formed by placing copper electrode tapes on top of the LCP. The substrate with the electrodes is attached to the drum collector and nanofibers are deposited by the electrospinning process for a period of 30 min to form a thick enough layer of nanofibers. This attachment method in depositing nanofibers onto the substrate to form the sensor represents a direct-write method which does not require the process of transfer of PVDF nanofibers onto the substrate [51]. The sensor development process is shown in Figure 2a and the schematic of the final sensor with a thin layer of transparent polyamide protective sheet is shown in Figure 2b.

The structure and the composition of the different phases of the polymorphic PVDF nanofibers after electrospinning is obtained by Fourier-transform infrared (FTIR, Nicolet 6700, Thermo Scientific, Waltham MA, USA) spectroscopy. The surface morphology of the nanofiber samples is obtained using Field Emission Scanning Electron Microscope (FESEM, JEOL Ltd., Tokyo, Japan). The frequency of different sizes of nanofibers present in the sample is obtained using Image J which is an open-source image processing software.

The vibration and strain inputs are provided using a mini shaker controlled by a frequency generator and amplifier (Data Physics, San Jose, CA, USA). A laser displacement sensor (LDS) (Keyence Corporation, Osaka, Japan) is used to compare the frequency and strain readings of the sensor. A Babolat 19 Gauge string (Lyon, France) is used for the string testing. The string is subjected to the required tension using a spring weight machine.

## 3. Results and Discussion

The following sections describe the results of the composition study of PVDF, frequency and strain characterization of the sensor in cantilever mode and single impact and sinusoidal vibration testing of the sensor attached to the string.

### 3.1. FTIR and DSC Characterization for β-phase Composition

PVDF is a polymorphic polymer which can be found in five phases, α, β, γ, δ and ε phase [52]. The naturally occurring phase is the α-phase which is the constitutive phase in the raw powder of PVDF. The electrospinning of the PVDF solution in acetone and DMF, converts the α-phase into the β-phase, due to the stretching of the nanofibers under a high electric voltage. The β-phase is the phase that has the highest contribution to the piezoelectricity of PVDF. Thus, the voltage output of the nanofibers formed by electrospinning is greater for samples with a larger β-phase fraction [53]. The amount of α-phase that gets converted to the β-phase depends on the parameters of electrospinning which need to be optimized for a given electrospinning system for better voltage output from the nanofibers. The effect of voltage on the fraction of β-phase in electrospun PVDF has been studied in detail in previous studies and it was shown that at the electrospinning voltage value of 15 kV, the electrospinning sample had the maximum β-phase fraction [53]. Hence, this voltage value is chosen for the present study for electrospinning. As for spinning distance, an increase in the distance allows for the electrospun nanofibers to stretch more and provides sufficient time for the solvents, in this case, DMF and acetone, to evaporate. However, further increase in the spinning distance caused jet instability and random deposition of the nanofibers. Hence, a mid-range value of 15 cm is chosen as the spinning distance for nanofiber deposition. For the collector drum speed, at very low drum speeds the fiber formation speed is higher than the drum speed and hence causes the fibers to entangle with each other while at higher drum speed the diameter of the nanofiber increases without any effect on the β-phase fraction of the sample [54,55]. Hence, the RPM of the drum collector, which can be varied between 100 and 1900 for the current set-up, is kept at a mid-range value of 1000. The aim of this set of experiments is to study the effect of needle size and flow rate on the electrospinning of the PVDF nanofibers and their effect on the β-phase of the nanofibers formed by keeping other parameters at a constant value. The range of values for flow rate and needle size for comparison has been chosen which have shown to produce fibers without beads in previous studies on PVDF nanofibers [56,57].

**Figure 3:**
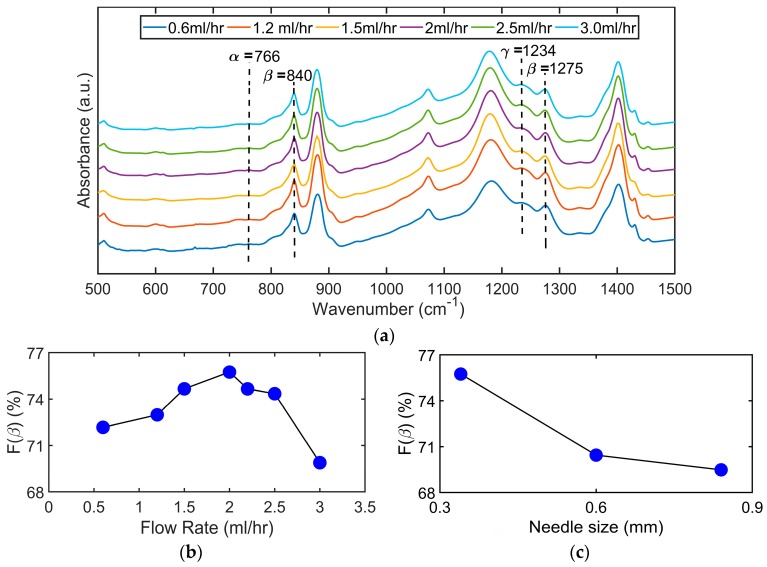
FTIR absorbance data of PVDF nanofibers prepared by electrospinning: (**a**) FTIR plot of absorbance for different flow rates. The peaks at 840 and 1275 cm^−1^ denote the presence of β-phase in the sample; (**b**) Fraction of β-phase (in percentage) present in the sample against the flow rate; (**c**) Fraction of β-phase (in percentage) present in the sample against three different needle size.

Three different sizes of needles with internal diameters of 0.84, 0.60 and 0.34 mm (18 G to 23 G) are used to form the nanofibers through electrospinning. Seven different flow rates ranging between 0.6 mL/h and 3.0 mL/h are used. The corresponding FTIR plots for the electrospun samples for different flow rates are shown in Figure 3a. The peak at 1275cm^−1^ corresponds to the β-phase which is the main contributing phase to the piezoelectricity of PVDF. The peak at 840 cm^−1^ signifies β-phase, however, it is debated in the literature that this peak also belongs to γ-phase [58]. The larger magnitude of the β-phase peak compared to the α-phase peak at 766 cm^−1^ signifies the dominance of β-phase in the electrospun nanofibers. This occurs due to the stretching under high voltage applied in the electrospinning process, as the raw powder consists purely of α-phase. The β-phase content in the electrospun nanofibers can be calculated from the FTIR spectra with the assumption that the absorption spectrum follows the Beer-Lambert law. The absorption coefficients were calculated by Gregorio [59] at the respective wavenumbers corresponding to the α-phase and the β-phase which are 766 and 840 cm^−1^. The relative fraction of β-phase in the sample is given by the following formula [59]:

(1) $$F\left(\beta \right)=\frac{A_{\beta }}{\left(\frac{K_{\beta }}{K_{\alpha }}\right)A_{\alpha }+A_{\beta }}$$

where *F*(*β*) represents the fraction of β-phase in the sample; *A_α_* and *A_β_* correspond to the absorbance values at 766 and 840 cm^−1^ and *K_α_* and *K_β_* are the absorption coefficients at 766 and 840 cm^−1^. The absorption coefficients values were given as 6.1 × 10^4^ and 7.7 × 10^4^ respectively [59]. The corresponding percentage of β-phase for different flow rates and needle size are shown in Figure 3b,c, respectively.

The effect of flow rate and needle size is initially studied by the use of two needle sizes of 0.84 mm and 0.34 mm and flow rates of 0.6 mL/h and 1.2 mL/h. The results show a higher β-phase percentage at the lower needle size of 0.34 mm and a higher flow rate of 1.2 mL/h. The effect of flow rate is then calculated by keeping the needle size fixed at 0.34 mm and increasing the flow rate up to 3 mL/h. It is seen in Figure 3b that the percentage of β-phase is increased with the increase in flow rate at lower values of flow rate till a flow rate of 2.0 mL/h and then starts to reduce for higher values. These results are in support with the some of the literature in which the β-phase percentage was seen to increase with flow rate at lower values of flow rates [57] while the results are in contrast to some research in which there was no or negative effect of flow rate shown on β-phase fraction [60]. The effect of needle size is calculated by keeping the flow rate fixed at 1.2 mL/h and use of three different needles with internal diameters of 0.84 mm, 0.6 mm and 0.34 mm. The results are shown in Figure 3c in which the β-phase percentage fraction is seen to increase as the needle size is reduced. These results seem to be in agreement with the study by Jiyong et al. [56]. The initial increase in β-phase percentage due to increase in flow rate for different needles can be attributed to the shear force exerted by the needle on the viscous solution however it also causes flow instability and has been linked to formation of beads in the electrospun nanofibers. The flow instability is seen at the lower flow rate of 0.6 mL/h but improved when it is increased to 1.2 mL/h. At very high flow rates, the solution doesn’t get enough time to evaporate due to the high rate of formation of nanofibers and hence the stretching of the jet is reduced. As a result, the poling stretch of the nanofibers is reduced and hence the percentage of β-phase conversion reduces in the electrospun nanofibers for higher flow rates. The increase in the β-phase with the reduction in the needle size from 0.84 mm to 0.34 mm can be explained by the higher shear force exerted by the smaller needles which aid in jet stretching.

Besides the larger fraction of β-phase for good piezoelectric response from the nanofiber web, the crystallinity or the preferential poling of the piezoelectric material is also an important factor that establishes whether the piezoelectric material will produce sufficient piezoelectric response to a stimulus such as strain. For this purpose, a differential scanning calorimetry (DSC) was performed for different nanofiber samples. It is also to be noted that according to one of the studies, it was reported that electrospinning of nanofibers with a rotating drum collector does provide a preferential poling for the CF_2_ dipoles. Hence, there is no need for any additional poling for PVDF nanofibers electrospinning as is required in the case of the PVDF films [61]. The DSC was done on a Q 200 DSC purchased from TA Instruments (New Castle, DE, USA). It was conducted at a heating rate of 10 °C/min from room temperature to 250 °C under a nitrogen flux of 50 mL/min. The variation in the percentage crystallinity of the electrospun nanofibers is investigated, over a range of flow rates and needle sizes. The degree of crystallinity (*X_c_*)for a given sample can be determined from its enthalpy of melting by the following relation:

(2) $$X_{c}\left(\%\right)=\frac{\Delta H_{m}}{\Delta H_{0}}\times 100$$

where Δ*H_m_* the melting enthalpy of the material and Δ*H*_0_ is the enthalpy of melting of the perfectly crystalline form of the material.

**Figure 4:**
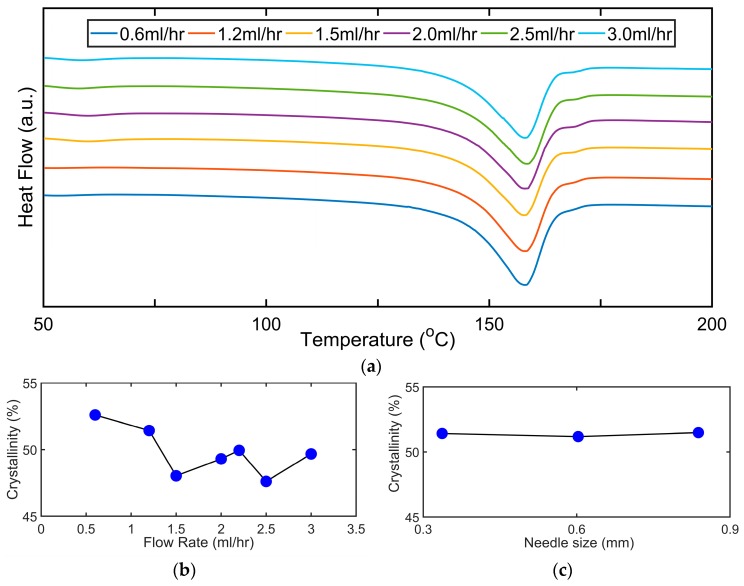
DSC characterization of various samples made with different flow rates and needle sizes: (**a**) the DSC thermograms for nanofibers obtained from different flow rate for the electrospinning process, the melting point was approximately around 158 °C for the nanofiber samples; (**b**) the variation of the crystallinity for different flow rates; (**c**) the variation of the crystallinity for different needle sizes employed for electrospinning.

To determine the degree of crystallinity in this paper, the value of Δ*H*_0_ for PVDF is taken as 104.7 J/g [62]. The DSC plots for the first heating cycle of the sample for the different flow rates of electrospun PVDF nanofibers samples are shown in Figure 4a. The peak temperature of the endothermic curve for the samples was found to be around 158 °C which is consistent with a previous reported finding [63]. A small endothermic peak is seen around 54 °C for all the samples which is said to be indicative of an upper glass transition temperature and associated with the *α_c_* form [64]. Although the polymorphic forms of PVDF can sometimes be deduced from the DSC thermograms, certain research works had advised against it because of the closeness of the melting point temperatures of the α and β phases [65].

On the variation of *X_c_* with flow rate, this is shown in Figure 4b and variation in needle size is shown in Figure 4c. *X_c_* is seen to slightly reduce with an increase in the flow rate (0.6–3.0 mL/h). The slight reduction in the *X_c_* values, with an increase in the flow rate, can be attributed to the reduced solvent evaporation at higher flow rates. This reduces the effective stretching of the nanofibers between the nozzle and the collector under a constant electric field which can lead to a reduction in the degree of crystallinity of the nanofibers. This is in agreement with [56] where the authors found that the crystallinity increased in the range of (0.5–1.0 mL/h) but reduced with a further increase in flow rate (1–3.0 mL/h).

For the variation of *X_c_* with needle size as shown in Figure 4c, the *X_c_* is observed to be almost constant with the needle size ranging from (0.34–0.84 mm). The ineffectiveness of the size of the needles on the crystallinity of the sample suggests that the shear force exerted by the capillary does not affect the chain orientation of the dipoles. This result is in contrast to [55] where a slight increase was seen in *X_c_* values with a reduction in needle diameter.

It has been shown in the past by various researchers that a larger percentage of β-phase in the FTIR calculations and a high degree of crystallinity leads to a larger voltage output hence the flow rate of 2.0 mL/h and a needle size of 0.34 mm is used to obtain nanofiber samples in further vibration sensor fabrication. These parameters are also utilized because the electrospinning jet is found to be continuous and process flow was smooth during electrospinning. The different compositions used for the preparation of the samples and their corresponding β-phase percentage and thermal properties have been summarized in Table 1.

### 3.2. Vibration Frequency Detection by the Sensor in Cantilever Configuration

**Figure 5:**
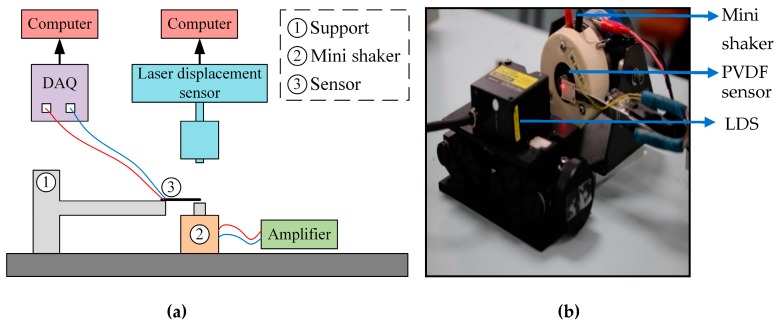
Experimental set-up for the vibration characterization of the sensor in cantilever configuration: (**a**) The schematic of the set-up shows the sensor attached to the support in a cantilever configuration and the corresponding equipment for the vibration testing; (**b**) A digital photo of the experimental set-up that shows the laser light from the LDS at the tip of the sensor.

The sensor is first subjected to several frequencies in the cantilever configuration and tested against a commercial laser displacement sensor (LDS) system to characterize its vibration response. The schematic of the experimental set-up is shown in Figure 5a and a digital photograph is shown in Figure 5b. The sensor is fixed at one end to a rigid support and the other end is free, hence resulting in a cantilever configuration. A mini shaker connected to an amplifier and frequency generator is used to test the sensor for different frequencies and amplitudes of the displacement. The vibration of the mini shaker results in displacement of the free end of the sensor, hence generating corresponding strain in the PVDF nanofibers. The (LDS) is used to measure the tip displacement of the free end of the cantilever sensor. The voltage output from the sensor is measured by an analog input data acquisition system (DAQ). The laser displacement sensor and the DAQ are connected to computer systems to enable the timestamps.

**Figure 6:**
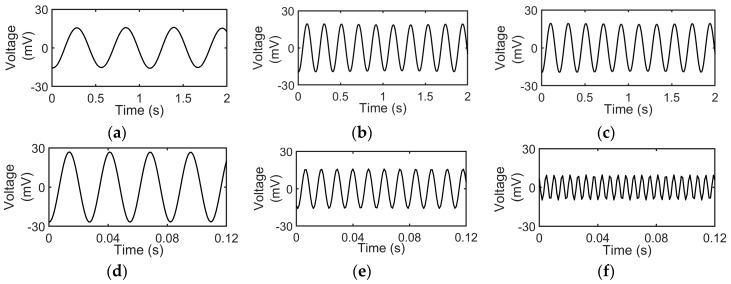
(**a**–**f**) Sensor voltage output to mini shaker amplitude perturbations at frequencies of 2 Hz, 5 Hz, 10 Hz, 40 Hz, 90 Hz and 180 Hz respectively.

The results of vibrating the sensor with six different frequencies are shown in Figure 6a–f. Each test is repeated three times or more. The frequency range is chosen such that it lies within the vibrational natural frequency experienced by the racket string after being impacted by a ball [66] or during the playing of a guitar which is at around 200 Hz [67]. The frequency generator produced a sinusoidal signal that drives the mini shaker. The voltage output from the PVDF sensor is a sinusoidal wave as can be seen from Figure 6a–f. It can be seen that the output of the sensor increases with frequency for Figure 6a–d which correspond to the frequency values of 2, 5, 10 and 40 Hz, respectively. This increase in the voltage output with the frequency is attributed to the increase in strain rate. The higher frequency of the mini shaker results in the reduction of the time taken between two subsequent stretch and release cycles of the cantilever than at a lower frequency. This means that the same amount of strain is applied to the sensor in a lesser amount of time and hence generates a higher strain rate. Thus, it can be deduced that the voltage output is also influenced by strain rate beside strain. This increase in the voltage output of the sensor with the strain rate in PVDF nanofibers has been reported previously [68,69]. The amplitude of the mini shaker vibrations is tried to be maintained at a constant value for all the frequency tests. However, after a frequency of 40 Hz, the amplitude of the mini shaker starts to decrease sharply. This is because the power of the mini shaker is proportional to its frequency and amplitude. After a frequency of 40 Hz, the mini shaker is not able to provide the required amplitude of displacement even at the highest displacement setting. Hence, it can be seen in Figure 6e,f that the voltage output has decreased as compared to other frequencies. This is due to reduced displacement and hence a reduction in strain in the PVDF nanofibers.

**Figure 7:**
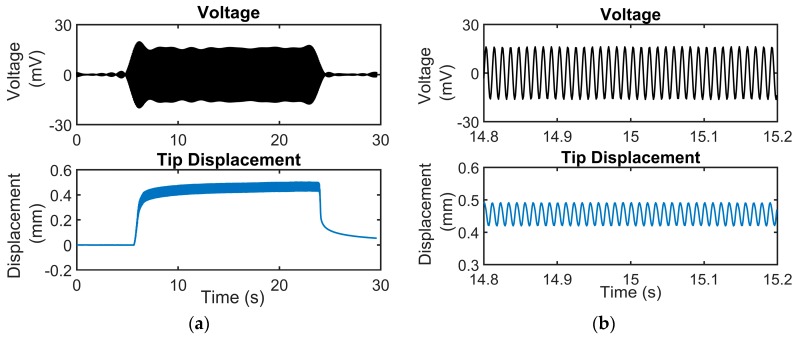
Voltage output from the sensor plotted against the tip displacement of the cantilever on the same scale for a frequency of 90 Hz: (**a**) Voltage output from the sensor against the tip displacement for a total testing duration of 30 s where the mini shaker is on between 5 and 25 s; (**b**) A zoomed in version of the voltage and tip displacement plot which shows the waveforms of the strain input and the corresponding voltage output of the sensor.

The voltage output of the sensor corresponding to its tip displacement at a frequency of 90 Hz is shown in Figure 7. The mini shaker is turned on after 5 s from the start of data acquisition and is continued to vibrate till 25 s after which it is turned off. The tip displacement is zero for the first 5 s as well as from 25 s to 30 s and hence there is no voltage output from the sensor corresponding to no change in strain. There is a corresponding voltage output from the sensor for the time the mini shaker is on. A zoomed in view of the plot shows that the tip displacement and the voltage output are both sinusoidal curves. This also seems to suggest that a repeatable voltage output can be obtained from the sensor for tip displacements.

The comparison between the frequency measured by the PVDF sensor and that measured by the commercial LDS sensor for the frequency plots shown in Figure 6 has been summarized in Table 2. The first column represents the reading of the manual knob for the frequency generator. The next two columns depict the frequencies obtained from the Fast Fourier Transform (FFT) of the voltage output of the sensor and the displacement output from the LDS. The results show that the sensor can predict the frequency within ±0.2% of the value measured by the LDS.

### 3.3. Strain Characterization of the Sensor

The second set of tests involves subjecting the proposed sensor to a range of tip displacement amplitude profiles ranging from 0.4–2.7 mm. The range of tip displacement amplitude is chosen as the span of deflection experienced near the end of a taut string at the edge of the racket frame considering a maximum displacement of 30 mm at the string centre. The range of strain was also suggested in other research work such as Maeda [70] where the highest peak to peak value of strain in a string was reported as 1300 με. The range of strain was reported as between 350 to 1300 με. Different values of tip displacements for the sensor are obtained by subjecting the sensor to mini shaker inputs at different amplitudes for less than 1 Hz frequency. The tip displacement values can be adjusted and controlled by adjusting the output amplitude of the signal generator. The tip displacement in this experiment is also measured using the LDS system. The tip displacement is then converted into corresponding strain value in the PVDF nanofibers by using cantilever relations. The strain experienced by the nanofibers due to a vertical tip displacement *h* of the free end of the cantilever is given as:

(3) $$\varepsilon =\;\frac{3yh}{2L^{2}}$$

where *ε* is the strain in the nanofibers, *y* is the height above or below the neutral axis and *L* is the total length of the cantilever.

**Figure 8:**
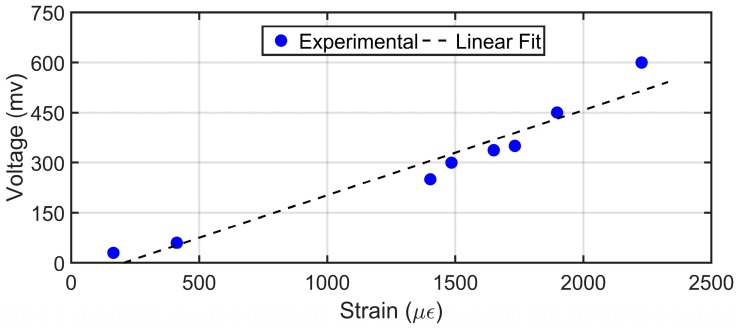
Relationship between voltage output and strain induced with different tip displacement in the PVDF sensor in cantilever configuration.

The results from the tests are shown in Figure 8. The plot shows a linear relationship between the voltage output and the applied strain due to the tip displacement provided to the sensor in the cantilever configuration. The sensitivity of the sensor to strain inputs was calculated to be 0.2 mV/με for the range of strain between 200 to 2300 με.

### 3.4. Sensor Attachment on String for Impact Detection and Frequency Measurement

The characterization of the sensor for frequency and strain in cantilever mode is necessary to understand the behaviour and the performance of the sensor. The tests are also necessary to determine the suitability of the sensor in the working range required for the applications mentioned in Section 1. This section discusses the performance of the sensor on attachment to a string when subjected to impact and sinusoidal vibrations.

**Figure 9:**
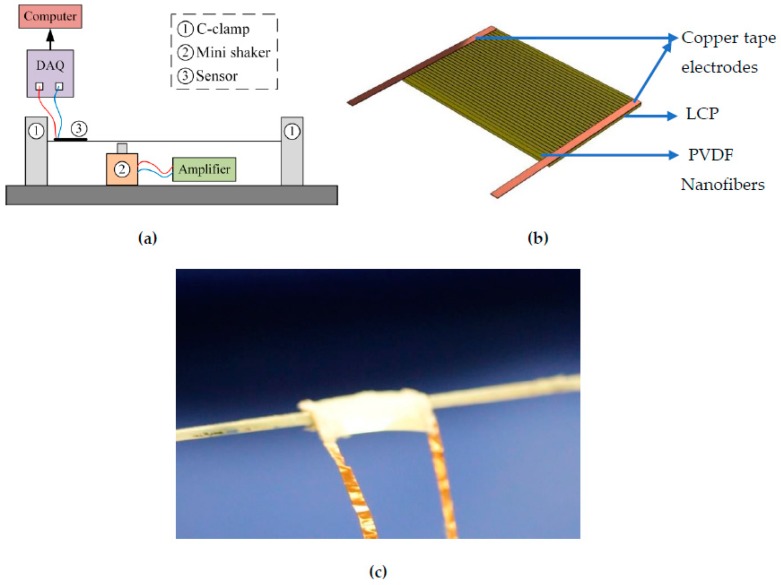
(**a**) Schematic of the sensor on string testing set-up; (**b**) Schematic of the developed sensor with electrospun PVDF nanofibers on top; (**c**) Close up digital photo of the sensor attached to the string.

A racket string is fixed between two C-clamps with a tension of 40 lbs achieved by pulling the string with a spring weight scale to the required tension value and clamping. The PVDF sensor is attached to the string with a resin-based glue and left overnight for the bond to strengthen. The output from the sensor is connected to a data acquisition system for recording the voltage output obtained from the sensor. The data acquisition is controlled by a computer connected to the DAQ unit. The mini shaker connected to a signal generator and amplifier is used to provide a sinusoidal displacement to the string. The mini shaker can be replaced with another object such as a hammer to characterize an impact on the string. The schematic of the experimental set-up is shown in Figure 9a. The placement of copper tape electrodes is changed to suit the attachment of the sensor on the string and the direction of the electrospun nanofibers is kept parallel to the string length direction. The schematic of the sensor is shown in Figure 9b and a close-up view of the sensor attached on the string is in Figure 9c.

#### 3.4.1. Single Impact Hammer Vibration Testing of the Sensor on String

**Figure 10:**
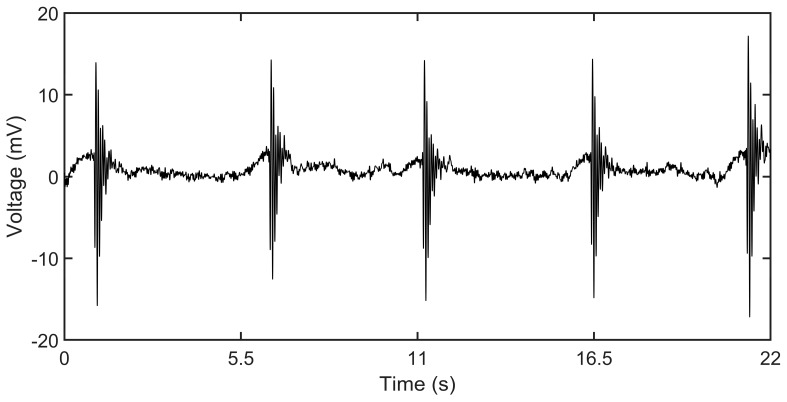
Sensor voltage output of the impact hammer on the string.

An impact hammer test is first done to examine the performance of the sensor on a single impact force subjected to the string. The string is struck at the center with a light hammer constituting of a flat tip at intervals of around 5 s. The output of 5 such hits with the hammer is shown in Figure 10. The peak to peak output voltages vary between 25–35 mV and the output shows a repeatable voltage profile. The hammer impact on the string generates a very short cycle of stretch and release, causing the string to vibrate which produces the corresponding voltage output profile generated by the piezoelectric PVDF nanofiber sensor. The vibration quickly reduces to zero due to the damping produced by the clamping of the string to the fixed iron C-clamps.

#### 3.4.2. Sinusoidal Vibration Testing of the Sensor on String

**Figure 11:**
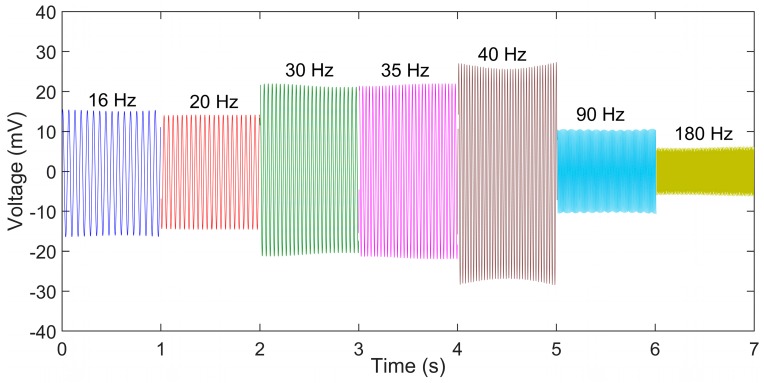
Voltage output response of the sensor to sinusoidal displacement input to the string.

The string is then subjected to a vibrational impact provided by the mini shaker and the output of the sensor is recorded. The output performance of the sensor is shown in Figure 11 against different frequencies.

The output voltage generated by the sensor varied from 10 mV for 180 Hz frequency to 50 mV peak to peak for a frequency of 40 Hz. The voltage output follows similar trend to the cantilever testing pattern with the output increasing with the frequency till 40 Hz and then dropping due to reduction in displacement amplitude as explained in Section 3.2. It can be observed that the output of the sensor for the same amplitude of strain and frequency for the string is less than in the cantilever configuration. There can be a few factors that can affect this. First, the size of the sensor attached to the string is less than that of the sensor tested under cantilever configuration. Hence the number of the nanofibers stretched in the string attachment is lesser than the cantilever configuration.

Secondly, the interface between the string and the sensor could lead to a reduction in the overall strain experienced by the sensor. The results in Figure 11 show the repeatable nature of the sensor to predict the strain produced in the string. The hammer test is done to understand the performance of the sensor in application with impact on the string. Sports rackets are one such area where the ball makes an impact on the string and leaves. The results of the developed sensor show that it can be used for impact characteristics measurement in strings.

## 4. Conclusions

A self-powered, flexible and light-weight PVDF nanofiber sensor on LCP substrate, that is capable of accurately measuring the vibration frequency and strain in the given range of applications has been developed. The sensor’s characteristics of being light-weight and self-powered makes it suitable for miniaturization. These characteristics enable its attachment to a string and measurement of the vibration characteristics of an impact force as well as a periodic force on the string.

PVDF nanofibers were formed with the use of electrospinning technique. The characterization of the nanofibers was done for morphology using FESEM which showed fibers were formed without any beads and the mean diameter of the fibers was (218 ± 53 nm). The percentage of β-phase was optimised between three different needle sizes of 0.84, 0.60 and 0.34 mm and flow rates ranging between (0.6–3.0 mL/h). Since, for electrospun nanofibers in general, a higher β-phase leads to a higher voltage output from the nanofibers, the needle size of 0.34 mm and flow rate of 2.0 mL/h was used for sensor development.

The experimental results of testing the sensor in the cantilever configuration showed a good agreement between the developed PVDF sensor and a commercial LDS system in identifying vibration frequencies between 2 to 180 Hz (± 0.2%). The output voltage of the sensor was seen to vary with the strain as well as the frequency of strain or the strain rate.

For a free cantilever motion, the sensor readings show a linear relationship between voltage output and strain (0.2 mV/με) in the range of strain between 200 to 2300 με. This indicates the suitability of the sensor for the range of applications involving a string.

The attachment of the developed PVDF sensor on a racket string was successfully achieved and the sensor showed a good response to vibration testing on the string. The results from a single impact hammer force on the string showed a repeatable voltage output of 25–35 mV from the sensor obtained with a light hit. The sensor also produced repeatable voltage output on the application of sinusoidal displacement of the string with a mini shaker. The development of a sensor that can be attached to a smaller curved surface like a string, opens a new area in the miniaturization of flexible sensors in the series of smart devices for IOT.

## Figures and Tables

**Table 1:** Effect of flow rate and needle size on β-phase percentage and thermal properties of PVDF nanofibers.

Sample	Flow Rate (mL/h)	Needle (nozzle) Size Gauge (mm)	F(β) (%)	Peak Temperature, *T_mp_* (°C)	Δ*H_m,_* Melting Enthalpy (J/g)	*X_c_* (%)
A	0.6	18 G (0.84)	66.9	158.14	53.53	51.1
B	0.6	23 G (0.34)	72.2	158.04	55.07	52.6
C	1.2	18 G (0.84)	69.5	158.24	53.91	51.5
D	1.2	20 G (0.6)	70.4	158.03	53.59	51.2
E	1.2	23 G (0.34)	73.0	157.94	53.84	51.4
F	1.5	23 G (0.34)	74.7	157.75	50.29	48.0
G	2.0	23 G (0.34)	75.8	157.71	51.62	49.3
H	2.2	23 G (0.34)	74.6	157.63	52.29	49.9
I	2.5	23 G (0.34)	74.3	158.34	49.85	47.6
J	3.0	23 G (0.34)	69.9	157.85	52.01	49.7

**Table 2:** Comparison between frequency measured by the mini shaker, LDS and the developed PVDF sensor.

Frequency (Hz) Obtained by Mini–Shaker (Reading of the Manual Knob)	Frequency (Hz) Obtained by LDS	Frequency (Hz) by PVDF Sensor	Percentage Deviation in Frequency (Hz) Measured by PVDF Sensor Compared to the LDS
2	1.82 ± 0.01	1.82 ± 0.04	0
5	4.74 ± 0.01	4.73 ± 0.14	0.2
10	9.63	9.64	0.1
40	36.60	36.60 ± 0.01	0
90	89.92 ± 0.05	89.90	0.02
180	183.41	183.43	0.01
